# Antinociceptive Effect of* Tephrosia sinapou* Extract in the Acetic Acid, Phenyl-p-benzoquinone, Formalin, and Complete Freund's Adjuvant Models of Overt Pain-Like Behavior in Mice

**DOI:** 10.1155/2016/8656397

**Published:** 2016-05-12

**Authors:** Renata M. Martinez, Ana C. Zarpelon, Talita P. Domiciano, Sandra R. Georgetti, Marcela M. Baracat, Isabel C. Moreira, Cesar C. Andrei, Waldiceu A. Verri, Rubia Casagrande

**Affiliations:** ^1^Department of Pharmaceutical Sciences, Health Science Centre, University Hospital, Londrina State University, Londrina, PR, Brazil; ^2^Departamento de Patologia, Centro de Ciências Biológicas, Universidade Estadual de Londrina, Londrina, PR, Brazil; ^3^Universidade Tecnológica Federal do Paraná, Londrina, PR, Brazil; ^4^Laboratório de Pesquisa em Moléculas Bioativas, Departamento de Química, Universidade Estadual de Londrina, Londrina, PR, Brazil

## Abstract

*Tephrosia toxicaria*, which is currently known as* Tephrosia sinapou* (Buc'hoz) A. Chev. (Fabaceae), is a source of compounds such as flavonoids.* T. sinapou* has been used in Amazonian countries traditional medicine to alleviate pain and inflammation. The purpose of this study was to evaluate the analgesic effects of* T. sinapou* ethyl acetate extract in overt pain-like behavior models in mice by using writhing response and flinching/licking tests. We demonstrated in this study that* T. sinapou* extract inhibited, in a dose (1–100 mg/kg) dependent manner, acetic acid- and phenyl-p-benzoquinone- (PBQ-) induced writhing response. Furthermore, it was active via intraperitoneal, subcutaneous, and peroral routes of administration.* T. sinapou* extract also inhibited formalin- and complete Freund's adjuvant- (CFA-) induced flinching/licking at 100 mg/kg dose. In conclusion, these findings demonstrate that* T. sinapou* ethyl acetate extract reduces inflammatory pain in the acetic acid, PBQ, formalin, and CFA models of overt pain-like behavior. Therefore, the potential of analgesic activity of* T. sinapou* indicates that it deserves further investigation.

## 1. Introduction

*Tephrosia sinapou* (Buc'hoz) A. Chev., also named* T. toxicaria* Pers, is a shrub that has been used in Amazonian countries traditional medicine to alleviate pain and inflammation [1].* Tephrosia* (Fabaceae) is a large perennial genus, distributed in warm regions of both hemispheres [2] known by several important pharmacological activities such as analgesic [3, 4], anti-inflammatory [3–6], antioxidant [5–8], anticancer [9, 10], insecticidal [11], larvicidal [8], and antiviral activities [12, 13].

Besides cancer chemopreventive activity [10],* Tephrosia sinapou* also exhibits larvicidal activity against* Aedes aegypti*, the main vector of dengue fever [14]. Furthermore,* T. sinapou* extract presents antioxidant activity [5–8] and inhibits oxidative stress* in vitro* by scavenging free radicals, iron chelating activity, and inhibition of iron-dependent and iron-independent lipoperoxidation [5].* T. sinapou* extract also reduces inflammatory total leukocytes and neutrophil recruitment induced by a variety of inflammatory stimuli in mice by a mechanism related to inhibition of proinflammatory cytokine (TNF-*α* and IL-1*β*) production and in a nitric oxide dependent manner [5]. Moreover,* T. sinapou* extract inhibits inflammatory hyperalgesia in mice by activating an opioid receptor-dependent mechanism [3]. The antinociceptive and anti-inflammatory efficacy of* T. sinapou* in the model of zymosan-induced temporomandibular joint inflammatory hyperalgesia in rats depends, at least in part, on the integrity of the HO-1 pathway [4]. Importantly, this plant is effective and safe, since the therapeutic dose did not produce any signs of toxicity [4].* T. sinapou* is a source of flavonoids and rotenoids that possess various biological effects [3, 4, 10]. In fact, plant extracts containing flavonoids are reported to own antinociceptive, anti-inflammatory, and antioxidant activities [15–19].

Currently available animal models evaluate two main symptoms of pain: (i) overt nociception/overt pain or (ii) hyperalgesia. In the first, varied nociceptive stimuli induce declared behavior such as abdominal contortions (writhing) and paw flinch or licking without further mechanical or thermal external stimuli. This declared behavior occurs because the overt nociceptive stimuli activate or induce fast production of endogenous mediators that activate the primary nociceptive neurons. These stimuli are in general chemical such as acetic acid, phenyl-p-benzoquinone (PBQ), and formalin, and a mixture of chemical and biological agent such as complete Freund's adjuvant (CFA) [20–23]. The second (hyperalgesia) results from the sensitization of nociceptive neurons and to be detected needs further stimulation of the nociceptors with mechanical stimuli [21, 24]. Despite the demonstrated antihyperalgesic efficacy of* T. sinapou* in preclinical studies [3, 4], no study assessed the antinociceptive efficacy of* T. sinapou* in PBQ, formalin, and CFA tests which are widely used, easy to learn, replicable, and fast to perform models. Therefore, further evidence on the antinociceptive effect of* T. sinapou* is necessary to determine whether or not it inhibits inflammatory overt pain-like behavior. Thus, we evaluated the antinociceptive effects of* T. sinapou* ethyl acetate extract in overt pain-like behavior models in mice. Furthermore,* T. sinapou* antinociceptive effect was evaluated using varied routes of administration.

## 2. Materials and Methods

### 2.1. Animals

The experiments were performed on male Swiss mice (20–25 g) from Universidade Estadual de Londrina (Londrina State University, Londrina, PR, Brazil) housed in standard clear plastic cages in a temperature-controlled room (23 ± 2°C), 12 h light and 12 h dark cycles and access to water and food* ad libitum*. All testing was performed between 9 a.m. and 5 p.m. Animals' care and handling procedures were performed in accordance with National Institutes of Health guidelines for the welfare of experimental animals and with the approval of the Ethics Committee of the Universidade Estadual de Londrina (registered under the number CEUA 80/10, process number 31468.2010.84). All efforts were made to minimize the number of animals used and their suffering.

### 2.2. Drugs and Reagents

Drugs and reagents were obtained from the following sources: Complete Freund's Adjuvant, phenyl-p-benzoquinone, Tween 80, dimethyl sulfoxide (DMSO) from Sigma Chemical Co. (St. Louis, MO, USA), formalin and acetic acid from Merck (Darmstadt, Germany), morphine sulphate from Cristalia (Itapira, SP, Brazil), and indomethacin from Prodome Quimica e Farmaceutica (São Paulo, SP, Brazil).

### 2.3. Plant Material

*Tephrosia sinapou* specimen was cultivated and collected at the Instituto Agronômico de Campinas (Agronomic Institute of Campinas (IAC)), Sao Paulo, Brazil, by S. Myasaka. Identification was performed by A. K. Pastorek in June 2005, and a voucher specimen was deposited at IAC under the number IAC 17211.

### 2.4. Preparation of the Extract

*T. sinapou* roots were dried and ground with a knife mill and then the powder (8.9 kg) was submitted to cool exhaustive extraction with ethyl acetate during 10 days with cycles of 48 h. Ethyl acetate extract was exhaustively washed with methanol followed by evaporation (final yield 58.7 g) [3, 5]. NMR and mass spectral analysis were used for preliminary phytochemical characterization of* T. sinapou* ethyl acetate extract. Two novel compounds were also identified, a substituted benzaldehyde and a chalcone. Phytochemical analysis of* T. sinapou* ethyl acetate extract by NMR and mass spectral analysis showed the presence of flavonoids: (1) a novel biflavonoid named toxicarine, constituted by glabranine and 5-O-methylnitenin units; (2) flavanone: 7-O-methylglabranine; (3) rotenoids: tephrosin, rotenolone, deguelin, 6-oxo-6a,12a-dehydrodeguelin, 6-oxo-6a,12a-dehydro- a-toxicarol, 6a,12a-dehydrorotenone, rotenonone, and villosone; (4) flavanols: quercetol B and tephrowatsin A. Other identified compounds were as follows: (1) coumarins: 2,3-dihydro-p-coumaric acid; pterocarpan: flamichapparin B; (2). Total flavonoid content was determined using the AlCl_3_ colorimetric method [3].

### 2.5. Experimental Protocols

Mice received intraperitoneal (i.p.), subcutaneous (s.c.), or per oral (p.o.) treatment with* T. sinapou* ethyl acetate extract (1, 3, 10, 30, and 100 mg/kg) or vehicle (20% Tween 80 in saline) 30 min before inflammatory stimulus. Mice were not fasted before the oral treatment. Inflammatory stimuli doses were based on previously published works [21, 23, 25–27] and also determined in our laboratory in pilot studies. Writhing response was evaluated for 20 min after i.p. injection of acetic acid or phenyl-p-benzoquinone. Paw flinching and licking nociceptive responses were quantified during 30 min after formalin or CFA injection.

### 2.6. Writhing Response Tests

Acetic acid-induced and phenyl-*p*-benzoquinone (PBQ) writhing models were performed as previously described [21]. Acetic acid (0.8% v/v, diluted in saline, 10 mL/kg), PBQ (diluted in DMSO 2%/saline, 1890 *μ*g/kg), or vehicle was injected into mice's peritoneal cavities. Each mouse was placed in a 10 cm diameter glass cylinder and the intensity of nociceptive behavior was quantified by counting the total number of writhes occurring between 0 and 20 min after stimulus injection [28, 29]. The writhing response consisted of a contraction of the abdominal muscle together with a stretching of hind limbs. The intensity of the writhing response was expressed as the cumulative writhing score over 20 min. Different individuals administered each test, prepared solutions to be injected, and performed the injections.

### 2.7. Formalin Test

The number of paw flinches and time spent licking the paw were determined within 0–30 min after intraplantar injection of 25 *μ*L of formalin 1.5%, as previously described [20]. The testing period was divided in intervals of 5 min and clearly demonstrated the presence of the first and second phases, which are characteristic of the method [20]. Results were obtained for both the first (0–5 min) and second (15–30 min) phases [30].

### 2.8. Complete Freund's Adjuvant (CFA) Test

Overt-pain-like behaviors were determined by the number of paw flinches and time spent licking the stimulated paw measured within 0–30 min after intraplantar injection of 10 *μ*L of CFA. The amount of time spent licking and flinching the injected paw was considered as indicative of nociception. Results were expressed by the total number of flinches and time spent licking performed in 30 min [25].

### 2.9. Statistical Analysis

Results are presented as means ± SEM of measurements made on 6 animals in each group per experiment; all experiments were performed twice. Two-way analysis of variance (ANOVA) was used to compare the groups and doses at all times (curves) when the nociceptive responses were measured at different times after the stimulus injection. The analyzed factors were treatments, time, and time* versus* treatment interaction. When there was a significant time* versus* treatment interaction, one-way ANOVA followed by Tukey's *t*-test was performed for each time. On the other hand, when the nociceptive responses were measured once after the stimulus injection, the differences between responses were evaluated by one-way ANOVA followed by Tukey's *t*-test. Statistical differences were considered significant at *P* < 0.05.

## 3. Results

### 3.1. *T. sinapou* Ethyl Acetate Extract Inhibited Writhing Response in Mice

**Figure 1:**
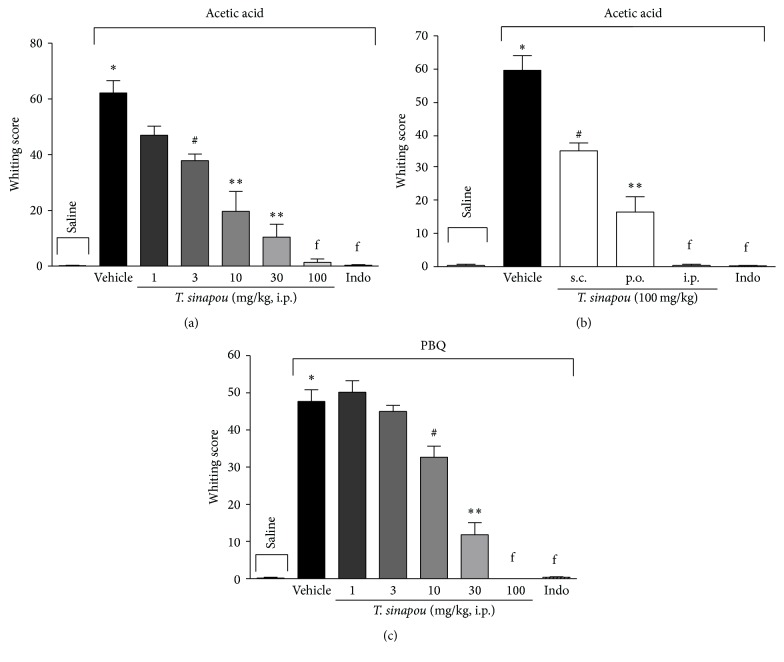
*T. sinapou* ethyl acetate extract inhibited writhing response in mice. Panel (a): mice were treated i.p. with* T. sinapou* ethyl acetate extract (1–100 mg/kg, 30 min), indomethacin (indo, 5 mg/kg, i.p. 40 min), or vehicle before i.p. stimulus with acetic acid (0.8% diluted in saline). Panel (b): mice were treated with 100 mg/kg of* T. sinapou* ethyl acetate extract (30 min) through i.p., p.o., and s.c. routes and indomethacin (indo, 5 mg/kg, i.p. 40 min) or vehicle. Panel (c): mice were treated i.p. with* T. sinapou* ethyl acetate extract (1–100 mg/kg, 30 min), indomethacin (indo, 5 mg/kg, i.p. 40 min), or vehicle before phenyl-*p*-benzoquinone (PBQ, 1890 *μ*g/kg diluted in 2% DMSO in saline) stimulus. Writhing score was evaluated during 20 min after stimulus injection. Experiments were performed with 6 mice per group per experiment and are representative of 2 independent experiments. ^*∗*^ *P* < 0.05 compared to the saline group, ^#^ *P* < 0.05 compared to the vehicle group, ^*∗∗*^ *P* < 0.05 compared to the vehicle group, the dose of 1 mg/kg (panels (a) and (c)), 3 mg/kg (panel (c)) of the extract, and s.c. route (panel (b)), and ^f^ *P* < 0.05 compared to the vehicle group and the doses of 1–10 mg/kg of extract (panel (a)), the doses 1–30 mg/kg (panel (c)), and s.c. and p.o. routes of administration.

A dose-dependent reduction of writhing response was observed with the doses of 3–100 mg/kg, i.p. of* T. sinapou* ethyl acetate extract but no effect with the lower dose of 1 mg/kg was observed (Figure 1(a)). The effect of 10 and 30 mg/kg doses was significantly greater than the 3 mg/kg dose, and the inhibition of acetic acid-induced writhing response by the dose of 100 mg/kg was significant compared to 10 mg/kg (Figure 1(a)). Therefore, the dose of 100 mg/kg of* T. sinapou* ethyl acetate extract was chosen for next experiments.

Extract treatment through different administration routes, i.p., s.c., or p.o., (Figure 1(b)), significantly inhibited acetic acid-induced writhings.* T. sinapou* ethyl acetate extract was effective to inhibit the writhing response induced by other stimulus such as PBQ at 1–100 mg/kg, i.p. (Figure 1(c)) in which significant inhibition was observed with doses greater than 3 mg/kg. In PBQ model,* T. sinapou* ethyl acetate extract inhibition by the doses of 30 and 100 mg/kg was significant compared to the lower doses of the extract. Indomethacin (5 mg/kg, i.p., 40 min) treatment inhibited the writhing response in all tests (Figure 1).

### 3.2. *T. sinapou* Ethyl Acetate Extract Inhibited Both Phases of Formalin-Induced Overt Pain-Like Behavior

**Figure 2:**
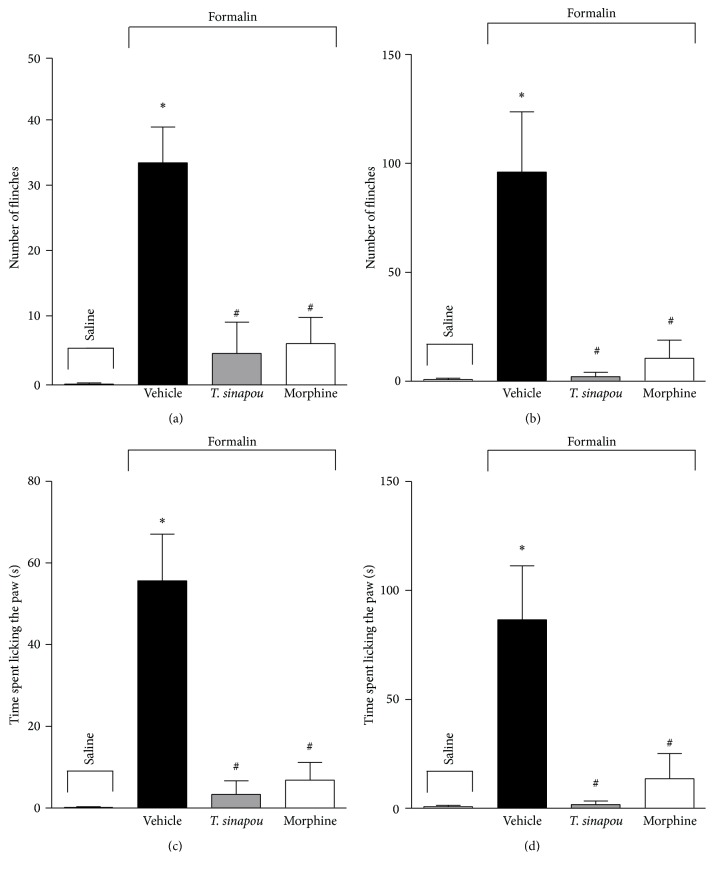
*T. sinapou* ethyl acetate extract inhibited both phases of formalin-induced overt pain-like behavior. Mice were treated with* T. sinapou* ethyl acetate extract (100 mg/kg, i.p., 30 min), morphine (5 mg/kg, i.p., 30 min), or vehicle before formalin injection. Total number of flinches of the first (panel (a)) and second (panel (b)) phases and time spent licking the paw of the first (panel (c)) and second (panel (d)) phases were evaluated at 0–5 min and 15–30 min intervals, respectively. Experiments were performed with 6 mice per group per experiment and are representative of 2 independent experiments. ^*∗*^ *P* < 0.05 compared to the saline group, ^#^ *P* < 0.05 compared to the formalin + vehicle group.

Pretreatment (30 min) with* T. sinapou* ethyl acetate extract (100 mg/kg, i.p.) significantly inhibited both phases of formalin-induced flinching (Figures 2(a) and 2(b)) and licking (Figures 2(c) and 2(d)). Morphine (5 mg/kg, i.p. 30 min) treatment inhibited both phases of formalin test (Figure 2).

### 3.3. *T. sinapou* Ethyl Acetate Extract Inhibited CFA- (Complete Freund's Adjuvant-) Induced Overt Pain-Like Behavior

**Figure 3:**
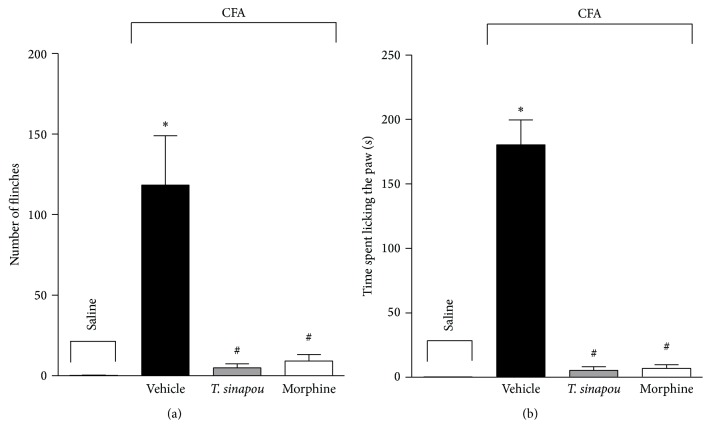
*T. sinapou* ethyl acetate extract inhibited CFA- (Complete Freund's Adjuvant-) induced overt pain-like behavior. Mice were treated with* T. sinapou* ethyl acetate extract (100 mg/kg, i.p., 30 min), morphine (5 mg/kg, i.p., 30 min), or vehicle before CFA (10 *μ*L/paw) injection. Total number of flinches (panel (a)) and time spent licking the paw (panel (b)) were evaluated for 30 min. Experiments were performed with 6 mice per group per experiment and are representative of 2 independent experiments. ^*∗*^ *P* < 0.05 compared to the saline group, ^#^ *P* < 0.05 compared to the formalin + vehicle group.

Pretreatment (30 min) with* T. sinapou* ethyl acetate extract (100 mg/kg, i.p.) significantly inhibited CFA-induced flinching (Figure 3(a)) and licking (Figure 3(b)). Morphine (5 mg/kg, i.p. 30 min) treatment inhibited CFA-induced overt pain-like behavior (Figure 3).

## 4. Discussion

We demonstrated that* T. sinapou* ethyl acetate extract inhibits inflammatory overt pain-like behavior in mice induced by chemical (acetic acid, PBQ and formalin) and chemical/biological (CFA) stimuli. The nociceptive behavior in these models depends on proinflammatory mediators such as cytokines is and susceptible to opioid treatment [21, 31]. Furthermore,* T. sinapou* extract was active through three different administration routes, such as i.p., p.o., and s.c.

Acetic acid and phenyl-p-benzoquinone (PBQ) models of nociceptive writhing response are simple and fast methods for novel drugs screening. Additionally, these methods involve complex mechanisms, including production of proinflammatory cytokines and opening of ion channels [21, 31]. Although acetic acid and PBQ induce similar behavioral responses, there are mechanistic differences between them. While acetic acid-induced writhing mechanism depends on peritoneal macrophages and mast cells activation which leads to the release of cytokines, such as TNF-*α* and IL-1*β* as well as sympathomimetic amines and eicosanoids [32], PBQ-induced writhing model depends on the cytokines IL-18, IFN-*γ*, and endothelin-1 [21]. On the other hand, the acetic acid and PBQ models share nociceptive mechanisms such as prostanoids, other cytokines like IL-33 [27], susceptibility to opioid treatment [21, 33], spinal cord mitogen-activated protein kinase, and phosphatidylinositol 3-kinase [23]. Since* T. sinapou* extract inhibited both models of writhing, it is likely that the antinociceptive action of* T. sinapou* extract relates to the common mechanisms in these models. Moreover, this finding is relevant since* T. sinapou* (100 mg/kg) was as effective as indomethacin, used as positive control.

It is noteworthy that all the chosen routes of administration were effective on inhibiting antinociceptive behavior. Nevertheless, there were significant differences in the antinociceptive effect of* T. sinapou* ethyl acetate extract depending on the route of administration. The descending order of activity was i.p., p.o., and s.c.. Considering that p.o. route effect was significantly greater than s.c. route, we speculate that it is a difference in the* T. sinapou* extract compounds pharmacokinetic rather than in the liability of its compounds in the gastric and intestinal tracts that modulated the antinociceptive effect of the extract. Oral delivery of drugs is regarded as the optimal route to achieve therapeutic effects against various diseases. Nonetheless, while oral route usually has maximum patient compliance, it presents poor bioavailability as a major issue to achieve the intended therapeutic responses [34]. Thus, we selected the administration route that allowed maximal effect of the extract to represent its maximal antinociceptive effect.

As mentioned above, formalin-induced overt pain-like behavior has two phases. The first phase (0–5 min after formalin) is the neurogenic phase and is generally attributed to a direct effect of the stimulus on primary nociceptive neurons, which depends on neurotransmitters such as serotonin, molecules from resident cells as histamine, and activation of TRPA1 (transient receptor potential ankyrin 1) receptors expressed by neurons [35]. The second phase (15–30 min after formalin) involves the subsequent development of inflammation, which is mediated by various cytokines, such as IL-33, TNF-*α*, IL-1*β*, IL-8, and IL-6, and prostaglandins [25, 27, 36–39]. Furthermore, there are some other important mechanisms in the formalin test such as dorsal root ganglia activation of the mitogen-activated protein kinase [40] and phosphatidylinositol 3-kinase [23, 41]. In this study, we demonstrated that* T. sinapou* ethyl acetate extract inhibited both phases of formalin test indicating that it prevents both neurogenic and inflammation processes development. The positive control drug morphine also inhibited formalin-induced nociceptive responses. Thus, the antinociceptive action of* T. sinapou* extract targets the mechanisms in this model.

It has been observed that CFA-induced mechanical and thermal hyperalgesia were reduced by TRPV1 antagonists or in TRPV1-deficient mice, and also CFA-induced increase of discharges of wide dynamic range neurons in response to thermal noxious stimulus was inhibited by TRPV1 antagonists [42, 43]. Therefore, TRPV1 mediates mechanical and thermal hyperalgesia induced by CFA. In CFA-induced overt pain-like behavior, pretreatment with* T. sinapou* also inhibited both paw flinching and licking behavior. Paw flinching behavior depends on peripheral and spinal nociceptive processing, while paw licking has the addition of supraspinal nociceptive structures [44, 45]. The present data on paw flinching and paw licking behaviors advance by showing that* T. sinapou* ethyl acetate extract may affect the peripheral, spinal, and supraspinal nociceptive processing involved in both the formalin test and CFA inflammation. Altogether,* T. sinapou* ethyl acetate extract inhibits extensively used models in preclinical studies searching for novel drugs and mechanisms of drugs. Therefore, inhibition in these models is an important finding consistent with conceivable applicability. Moreover,* T. sinapou* inhibited the zymosan-induced head withdrawal nociceptive threshold, the recruitment of inflammatory cells, myeloperoxidase activity, and temporomandibular joint immunohistochemical alterations by increasing HO-1 expression [4]. Additionally,* T. sinapou* did not show signs of toxicity when administered in subchronic toxicity protocol [4]. It is important to note that* T. sinapou* revealed the presence of flavonoids, including novel compounds [3], and these molecules are phenolic compounds where the analgesic activity has already been well demonstrated [26, 46–50].

## 5. Conclusions

In conclusion, the present study has demonstrated the antinociceptive activity of* T. sinapou* ethyl acetate extract in the models of acetic acid- and PBQ-induced writhing and formalin- and CFA-induced paw flinching and licking. The promising antinociceptive activity of* T. sinapou* [3, 4, and present data] indicates that it merits further preclinical and possible clinical investigation in pain.

## References

[1] Moretti C., Grenand P. (1982). Les Nivrées ou plantes ichtyotoxiques de la guyane française. *Journal of Ethnopharmacology*.

[2] Tarus P. K., Machocho A. K., Lang'at-Thoruwa C. C., Chhabra S. C. (2002). Flavonoids from *Tephrosia aequilata*. *Phytochemistry*.

[3] Martinez R. M., Zarpelon A. C., Cardoso R. D. R. (2013). Tephrosia sinapou ethyl acetate extract inhibits inflammatory pain in mice: opioid receptor dependent inhibition of TNF*α* and IL-1*β* production. *Pharmaceutical Biology*.

[4] do Val D. R., Bezerra M. M., Silva A. A. R. (2014). *Tephrosia toxicaria* Pers. reduces temporomandibular joint inflammatory hypernociception: the involvement of the HO-1 pathway. *European Journal of Pain*.

[5] Martinez R. M., Zarpelon A. C., Zimermann V. V. M. (2012). Tephrosia sinapou extract reduces inflammatory leukocyte recruitment in mice: effect on oxidative stress, nitric oxide and cytokine production. *Revista Brasileira de Farmacognosia*.

[6] Nile S. H., Khobragade C. N. (2011). In vitro anti-inflammatory and xanthine oxidase inhibitory activity of *Tephrosia purpurea* shoot extract. *Natural Product Communications*.

[7] Khan N., Sharma S., Alam A., Saleem M., Sultana S. (2001). Tephrosia purpurea ameliorates N-diethylnitrosamine and potassium bromate-mediated renal oxidative stress and toxicity in wistar rats. *Pharmacology and Toxicology*.

[8] e Vasconcelos J. N., Lima J. Q., de Lemos T. L. G. (2009). Estudo químico e biológico de *Tephrosia toxicaria* Pers. *Química Nova*.

[9] Saleem M., Ahmed S.-U., Alam A., Sultana S. (2001). *Tephrosia purpurea* alleviates phorbol ester-induced tumor promotion response in murine skin. *Pharmacological Research*.

[10] Jang D. S., Park E. J., Kang Y.-H. (2003). Potential cancer chemopreventive flavonoids from the stems of *Tephrosia toxicaria*. *Journal of Natural Products*.

[11] Andrei C. C., Ferreira D. T., Faccione M., de Moraes L. A. B., de Carvalho M. G., Braz-Filho R. (2000). C-prenylflavonoids from roots of *Tephrosia tunicata*. *Phytochemistry*.

[12] Andrei G., Snoeck R., Vandeputte M., De Clercq E. (1997). Activities of various compounds against murine and primate polyomaviruses. *Antimicrobial Agents and Chemotherapy*.

[13] Sánchez I., Gómez-Garibay F., Taboada J., Ruiz B. H. (2000). Antiviral effect of flavonoids on the dengue virus. *Phytotherapy Research*.

[14] Vasconcelos J. N. E., Santiago G. M. P., Lima J. Q. (2012). Rotenoids from *Tephrosia toxicaria* with larvicidal activity against Aedes aegypti, the main vector of dengue fever. *Quimica Nova*.

[15] Verri W. A., Vicentini F. T. M. C., Baracat M. M. (2012). Flavonoids as anti-inflammatory and analgesic drugs: mechanisms of action and perspectives in the development of pharmaceutical forms. *Studies in Natural Products Chemistry*.

[16] Campanini M. Z., Custódio D. L., Ivan A. L. M. (2014). Topical formulations containing pimenta pseudocaryophyllus extract: In vitro antioxidant activity and in vivo efficacy against UV-B-induced oxidative stress. *AAPS PharmSciTech*.

[17] Cascaes M. M., Guilhon G. M. S. P., de Aguiar Andrade E. H., das Graças Bichara Zoghbi M., da Silva Santos L. (2015). Constituents and pharmacological activities of *Myrcia* (Myrtaceae): a review of an aromatic and medicinal group of plants. *International Journal of Molecular Sciences*.

[18] Shah S. M. M., Shah S. M. H. (2015). Phytochemicals, antioxidant, antinociceptive and anti-inflammatory potential of the aqueous extract of *Teucrium stocksianum* bioss. *BMC Complementary and Alternative Medicine*.

[19] Rabelo A. S., Oliveira I. D., Guimarães A. G. (2013). Antinociceptive, anti-inflammatory and antioxidant activities of aqueous extract from *Remirea maritima* (Cyperaceae). *Journal of Ethnopharmacology*.

[20] Dubuisson D., Dennis S. G. (1977). The formalin test: a quantitative study of the analgesic effects of morphine, meperidine, and brain stem stimulation in rats and cats. *Pain*.

[21] Verri W. A., Cunha T. M., Magro D. A. (2008). Role of IL-18 in overt pain-like behaviour in mice. *European Journal of Pharmacology*.

[22] Magro D. A. C., Hohmann M. S. N., Mizokami S. S. (2013). An interleukin-33/ST2 signaling deficiency reduces overt pain-like behaviors in mice. *Brazilian Journal of Medical and Biological Research*.

[23] Pavao-de-Souza G. F., Zarpelon A. C., Tedeschi G. C. (2012). Acetic acid- and phenyl-p-benzoquinone-induced overt pain-like behavior depends on spinal activation of MAP kinases, PI_3_K and microglia in mice. *Pharmacology Biochemistry and Behavior*.

[24] Ferreira S. H., Nakamura M., de Abreu Castro M. S. (1978). The hyperalgesic effects of prostacyclin and prostaglandin E2. *Prostaglandins*.

[25] Mizokami S. S., Arakawa N. S., Ambrosio S. R. (2012). Kaurenoic acid from *Sphagneticola trilobata* inhibits inflammatory pain: effect on cytokine production and activation of the NO-cyclic GMP-protein kinase G-ATP-sensitive potassium channel signaling pathway. *Journal of Natural Products*.

[26] Borghi S. M., Carvalho T. T., Staurengo-Ferrari L. (2013). Vitexin inhibits inflammatory pain in mice by targeting TRPV1, oxidative stress, and cytokines. *Journal of Natural Products*.

[27] Magro D. A. C., Hohmann M. S. N., Mizokami S. S. (2013). An interleukin-33/ST2 signaling deficiency reduces overt pain-like behaviors in mice. *Brazilian Journal of Medical and Biological Research*.

[28] Calixto-Campos C., Carvalho T. T., Hohmann M. S. N. (2015). Vanillic acid inhibits inflammatory pain by inhibiting neutrophil recruitment, oxidative stress, cytokine production, and NF*κ*B activation in mice. *Journal of Natural Products*.

[29] Pinho-Ribeiro F. A., Zarpelon A. C., Fattori V. (2016). Naringenin reduces inflammatory pain in mice. *Neuropharmacology*.

[30] Ruiz-Miyazawa K. W., Zarpelon A. C., Pinho-Ribeiro F. A., Pavão-De-Souza G. F., Casagrande R., Verri W. A. (2015). Vinpocetine reduces carrageenan-induced inflammatory hyperalgesia in mice by inhibiting oxidative stress, cytokine production and NF-*κ*B activation in the paw and spinal cord. *PLoS ONE*.

[31] Verri W. A., Cunha T. M., Ferreira S. H. (2007). IL-15 mediates antigen-induced neutrophil migration by triggering IL-18 production. *European Journal of Immunology*.

[32] Ribeiro R. A., Vale M. L., Thomazzi S. M. (2000). Involvement of resident macrophages and mast cells in the writhing nociceptive response induced by zymosan and acetic acid in mice. *European Journal of Pharmacology*.

[33] Verri W. A., Cunha T. M., Poole S., Ferreira S. H., Cunha F. Q. (2007). Cytokine inhibitors and pain control. *Revista Brasileira de Reumatologia*.

[34] Pathak K., Raghuvanshi S. (2015). Oral bioavailability: issues and solutions via nanoformulations. *Clinical Pharmacokinetics*.

[35] McNamara C. R., Mandel-Brehm J., Bautista D. M. (2007). TRPA1 mediates formalin-induced pain. *Proceedings of the National Academy of Sciences of the United States of America*.

[36] Chichorro J. G., Lorenzetti B. B., Zampronio A. R. (2004). Involvement of bradykinin, cytokines, sympathetic amines and prostaglandins in formalin-induced orofacial nociception in rats. *British Journal of Pharmacology*.

[37] Parada C. A., Tambeli C. H., Cunha F. Q., Ferreira S. H. (2001). The major role of peripheral release of histamine and 5-hydroxytryptamine in formalin-induced nociception. *Neuroscience*.

[38] Puig S., Sorkin L. S. (1996). Formalin-evoked activity in identified primary afferent fibers: systemic lidocaine suppresses phase-2 activity. *Pain*.

[39] Verri W. A., Cunha T. M., Parada C. A., Poole S., Cunha F. Q., Ferreira S. H. (2006). Hypernociceptive role of cytokines and chemokines: targets for analgesic drug development?. *Pharmacology and Therapeutics*.

[40] Alter B. J., Zhao C., Karim F., Landreth G. E., Gereau R. W. (2010). Genetic targeting of ERK1 suggests a predominant role for ERK2 in murine pain models. *Journal of Neuroscience*.

[41] Pezet S., Marchand F., D'Mello R. (2008). Phosphatidylinositol 3-kinase is a key mediator of central sensitization in painful inflammatory conditions. *Journal of Neuroscience*.

[42] Honore P., Wismer C. T., Mikusa J. (2005). A-425619 [1-isoquinolin-5-yl-3-(4-trifluoromethyl-benzyl)-urea], a novel transient receptor potential type V1 receptor antagonist, relieves pathophysiological pain associated with inflammation and tissue injury in rats. *Journal of Pharmacology and Experimental Therapeutics*.

[43] Szabó Á., Helyes Z., Sándor K. (2005). Role of transient receptor potential vanilloid 1 receptors in adjuvant-induced chronic arthritis: in vivo study using gene-deficient mice. *Journal of Pharmacology and Experimental Therapeutics*.

[44] Porro C. A., Cavazzuti M., Lui F., Giuliani D., Pellegrini M., Baraldi P. (2003). Independent time courses of supraspinal nociceptive activity and spinally mediated behavior during tonic pain. *Pain*.

[45] Donahue R. R., LaGraize S. C., Fuchs P. N. (2001). Electrolytic lesion of the anterior cingulate cortex decreases inflammatory, but not neuropathic nociceptive behavior in rats. *Brain Research*.

[46] Valério D. A., Georgetti S. R., Magro D. A. (2009). Quercetin reduces inflammatory pain: Inhibition of oxidative stress and cytokine production. *Journal of Natural Products*.

[47] Hasanein P., Fazeli F. (2014). Role of naringenin in protection against diabetic hyperalgesia and tactile allodynia in male Wistar rats. *Journal of Physiology and Biochemistry*.

[48] Pinho-Ribeiro F. A., Hohmann M. S., Borghi S. M. (2015). Protective effects of the flavonoid hesperidin methyl chalcone in inflammation and pain in mice: role of TRPV1, oxidative stress, cytokines and NF-*κ*B. *Chemico-Biological Interactions*.

[49] Mohamad A. S., Akhtar M. N., Zakaria Z. A. (2010). Antinociceptive activity of a synthetic chalcone, flavokawin B on chemical and thermal models of nociception in mice. *European Journal of Pharmacology*.

[50] Rauf A., Khan R., Khan H., Ullah B., Pervez S. (2014). Antipyretic and antinociceptive potential of extract/fractions of *Potentilla evestita* and its isolated compound, acacetin. *BMC Complementary and Alternative Medicine*.

